# CogEvo, a cognitive function balancer, is a sensitive and easy psychiatric test battery for age‐related cognitive decline

**DOI:** 10.1111/ggi.13847

**Published:** 2019-12-18

**Authors:** Sadanobu Ichii, Takumi Nakamura, Takeshi Kawarabayashi, Masamitsu Takatama, Tetsuya Ohgami, Kazushige Ihara, Mikio Shoji

**Affiliations:** ^1^ Department of Social Medicine Hirosaki University Graduate School of Medicine Hirosaki Japan; ^2^ Department of Neurology Gunma University Hospital Maebashi Japan; ^3^ Department of Neurology, Dementia Research Center Geriatrics Research Institute and Hospital Maebashi Japan; ^4^ Department of Pharmacology Aomori University Aomori Japan; ^5^ Department of Neurobiology and Behavior Gunma University Graduate School of Medicine Maebashi Japan

**Keywords:** age‐related cognitive decline, cognitive function balancer, computer‐aided psychiatric test battery

## Abstract

**Aim:**

We examined whether a newly developed computer‐aided neuropsychiatric series of test, CogEvo, is necessary and sufficient for the evaluation of cognitive function in older people.

**Methods:**

A total of 272 participants in worthwhile life activity for the prevention of decline in mobility and cognitive function were administered tests every week at 33 locations in Fukaura‐machi, Japan. Basic profile information, a Mini‐Mental State Examination (MMSE), a CogEvo and a clock drawing test were used in the present study.

**Results:**

Our results are summarized as: (i) the total score of the CogEvo and MMSE tests decreased significantly according to age and in age group analysis; (ii) scores from the CogEvo and MMSE tests showed a significant correlation; (iii) MMSE scores showed marked ceiling effects; (iv) analysis of cognitive domains, such as orientation, attention, memory and executive function, and spatial cognition using CogEvo showed significant age‐dependent impairment; (v) CogEvo discriminated three score groups of MMSE results with sensitivity and specificity of 70% and 60% in the <23 score group, 78% and 54% in the 24–26 score group, and 85% and 70% in the >27 score group, respectively; (vi) CogEvo memory tests reflected more detailed recall function than registration function; and (vii) CogEvo spatial cognition test results were correlated with test items of the MMSE and clock drawing tests.

**Conclusions:**

CogEvo is an easy and potentially useful computer‐aided test battery that can be used to evaluate age‐related or pathological decline in cognitive function from middle age and in preclinical stages of dementia. **Geriatr Gerontol Int 2019; ••: ••–••**.

## Introduction

The number of dementia patients is rapidly increasing with the increasing older adult population in Japan. Cognitive impairment decreases life independence and impairs the quality of life in older adult populations. For dementia patients, medical therapy and home and social care intervention are necessary as well. These burdens are stressful for the family and caregivers, and are major factors in increasing national costs of medical care. Recent advances in dementia research have suggested that early detection of cognitive impairment and intervention are beneficial for dementia prevention, possible disease‐modifying therapy and development of social care systems for dementia patients. Pharmacists instruct patients to maintain adequate use of prescribed drugs from the pharmacy and home based on Japanese law. However, common prescriptions that are 90‐days long and polypharmacy of >6 drugs for elderly and dementia patients cause dropout from compliance, leading to low efficacy or adverse effects. Pharmacists should understand the cognitive function of people who are prescribed drugs easily and properly. To improve these situations, we hold educational campaigns about worthwhile life activity, including recollection, for the prevention of a decline in mobility and cognitive function in a salon for middle‐aged to elderly people at 33 locations in the Fukaura‐machi area, Aomori, Japan. Based on this activity, we examined whether a newly developed computer‐aided neuropsychiatric battery, “CogEvo, a cognitive function balancer”, is necessary and sufficient for the evaluation of cognitive function in older people.

## Methods

### *Participants*

A total of 272 participants were enrolled in this study. They consisted of 242 women and 27 men. The mean age was 79.5 ± 7 years, and the age range was from 40–97 years. They participated in worthwhile life activity for the prevention of decline in mobility and cognitive function in a salon for middle‐aged to elderly people. These activities took place every week at 33 locations in Fukaura‐machi, Japan. Approximately two to 20 people who participated in the activity were interviewed and examined after brief instruction on dementia and related disorders. The examination items were basic profile information on the participants, a Mini‐Mental State Examination (MMSE),^1^, ^2^, ^3^ a cognitive function balancer (CogEvo; Total Brain Care, Kobe, Japan) and a clock drawing test (CDT).^4^, ^5^, ^6^, ^7^ The mean examination times were 10 min in for the MMSE, 10 min for the CogEvo and 1 min for the CDT. This study was approved by the ethics committee of Hirosaki University (2017–1039). All participants provided written informed consent.

### *CogEvo, a cognitive function balancer*

CogEvo is a computer‐aided cognitive function test battery that uses a touch panel consisting of five basic tasks to evaluate orientation, attention, memory, executive function and spatial cognition. After audiovisual usage instruction, the participant pushes the start icon. If the answer is correct, 1 point is added. Incorrect answers are worth 0 points in five domain tasks. The scores for response time include four domain tasks, except the flashing light task. Each point is calculated according to the following formula: response time points = (standardized time limit – actual response time by tested participants) / standardized time limit × 100 points. The standardized time limit is the mean plus three standard deviations (3SD) of the time required for each task procedure based on preliminary trial data of CogEvo balancer for 50 000 participants. Total scores are the sum of both scoring systems.

#### *Orientation*

**Figure 1:**
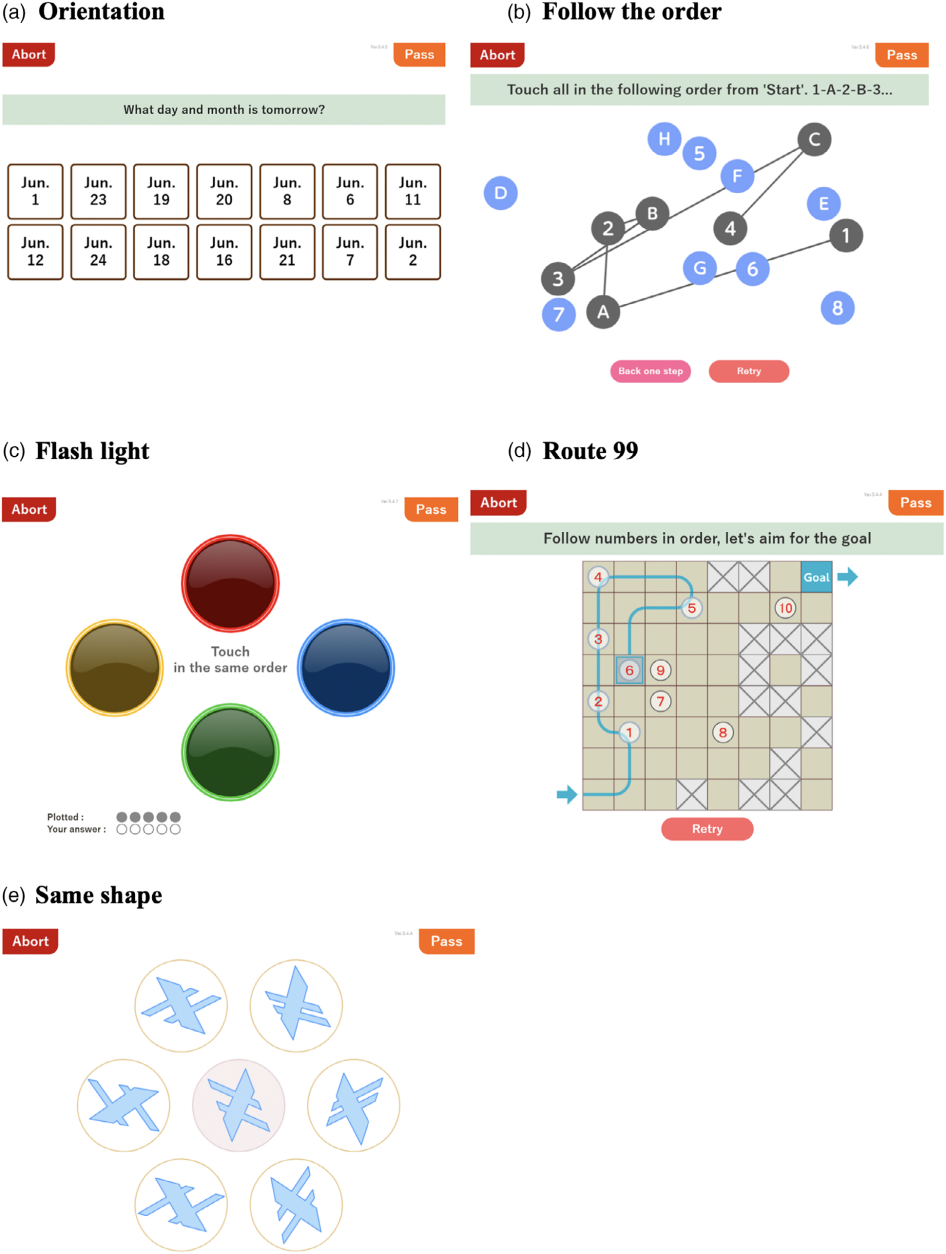
Cognitive function balancer (CogEvo).

The task of selecting the correct day, week and time of the examination day was included. Questions were randomly presented as 14 choices of days, seven choices of the week and 14 choices of time on a touch panel (Fig. 1a).

#### *Visual attention: Follow the order*

The purpose of the test was to touch numbers, Japanese hiragana or alphabet characters, and then touch both characters alternatively, on the panel according to their order. For example, the participant touched 1, 2, 3…, A, B, C, D… and 1, A, 2, B, 3, C, …. Each question consisted of following six digits, 12 characters and alternate combinations of eight digits with eight characters (Fig. 1b).

#### *Memory task: Flashing light*

After memorization of the random order of flashing red, blue, green and yellow lights, the participant touched the lights in the same order. Each light flashed for 1 s. In some cases, the same color light flashed in series. The task started with two lights flashing, and then the light number increased up to 16 flashing trials in the case of correct answers. Result points were calculated only as they related to accurate answering rates (Fig. 1c).

#### *Executive function: Route 99*

Participants traced squares on the panel from the start to the goal in sequence following randomly displayed digits in order from 1 to 10. Oblique passage or a route using the same square was prohibited. The task consisted of 16 squares (4 × 4), 36 squares (6 × 6) and 64 squares (8 × 8; Fig. 1d).

#### *Spatial cognition: Same shape*

The participants selected the same figure displayed on the center of the panel from six other figures around the center. A total of four questions consisted of randomly selecting seven figures from the total of 34 figures. The center figure and answer figure were in different locations, and at different rotation angles every time (Fig. 1e).

### *CDT*

The person was given a blank piece of paper and told to draw a clock that showed the time.^4^ Scoring was carried out based on 10 points by Rouleau^5^ except for prescribing the time as 10 min after 10 o'clock instead of 10 min after 11 oʼclock.

### *Statistical analysis*

Statistical analysis was carried out using linear regression analysis for correlation analyses, one‐way anova with Turkeyʼs multiple comparison tests for ordinal analyses, the Kruskal–Wallis test with Dunnʼs multiple comparison tests, an unpaired *t*‐test and a Mann–Whitney test after normality testing using GraphPad Prism, version 8 (GraphPad Software, San Diego, CA, USA). Receiver operating characteristic analyses were carried out using R Commander version 2.4.0 for windows (The Institute of Statistical Mathematics, Tokyo: https://cran.ism.ac.jp). Statistical significance was set at *P* < 0.05.

## Results

Basic profiles of participants are summarized in Table 1. We divided the total 272 participants into four groups according to age to analyze the age‐related natural course of cognitive function. The first group with participants aged 40–69 years included 21 participants (group 1), the next group had people who were aged 70–79 years and there were 105 in the group (group 2), the third group had people who were aged 80–89 year and there were 134 in the group (group 3), and the eldest group was aged 90–99 years and there were 12 people in the group (group 4). Groups 2 and 3 had enough participants for statistical analyses, but female‐dominant differences were observed in all groups. Total scores and subclassification domain items of the CogEvo, MMSE tests and the CDT are presented in Table 1. The mean scores for the total participants were 1001.1 ± 281.8 in the CogEvo tests, 25.8 ± 3.3 in the MMSE test and 7.7 ± 2.4 in the CDT, respectively. These scores declined with age.

**Table 1:** Baseline characteristics of participants

Participants	Total population	40–69 years	70–79 years	80–89 years	90–99 years	MMSE >27	MMSE 24–26	MMSE <23
No. participants	272	21	105	134	12	121	103	48
Mean age (years)	79.5 ± 7.0	64.6 ± 6.3	75.7 ± 2.5	83.7 ± 2.8	92.8 ± 2.3	77.4 ± 7.2	80.52 ± 6.1	82.94 ± 6.4
Female	246	20	96	118	12	107	95	44
Total score of CogEvo	1001.1 ± 281.8	1344.9 ± 216.9	1097.8 ± 231.4	893.4 ± 251.9	755.1 ± 246.5	1128.1 ± 248.4	954.6 ± 252.5	780.4 ± 252.2
Orientation	245.8 ± 86.2	315.7 ± 59.3	266.0 ± 77.1	223.2 ± 85.6	200.3 ± 92.8	277.4 ± 70.7	240 ± 80.8	178.8 ± 91.9
Follow the order	155.1 ± 50.1	212.1 ± 50.7	170.7 ± 43.6	137.9 ± 43.7	111.3 ± 38.4	176.5 ± 42.9	148.1 ± 47.8	116 ± 43.7
Flashing light	245.2 ± 122.8	338.6 ± 105.2	268.0 ± 110.7	218.4 ± 123.9	181.7 ± 103.1	283.3 ± 123.2	230 ± 111	181.7 ± 111.7
Route 99	130.9 ± 46.3	175.2 ± 38.2	138.3 ± 41.5	120.5 ± 45.6	105.0 ± 43.8	144.8 ± 46.1	122.9 ± 42.2	111 ± 45.1
Same shape	224 ± 90.7	303.3 ± 84.6	254.9 ± 78.1	193.4 ± 84.9	156.9 ± 76.0	246 ± 85.7	213.6 ± 87.2	190.8 ± 95.9
MMSE score	25.8 ± 3.3	27.7 ± 2.3	26.7 ± 2.6	25.3 ± 3.7	24.1 ± 2.9	28.5 ± 1.1	25.1 ± 0.8	20.6 ± 3.4
CDT	7.7 ± 2.4	9.1 ± 0.9	8.0 ± 2.2	7.4 ± 2.7	7.4 ± 3.0	8.5 ± 1.8	7.8 ± 2.3	5.8 ± 3.3

CDT, clock drawing test; MMSE, Mini‐Mental State Examination.

We also divided the total participants into three groups according to cut‐off scores for the MMSE; that is, 24 and 26 points, which are commonly used to discriminate dementia from normal individuals,^2^ and for registration criteria of the cognitively unimpaired (CU),^8^, ^9^ mild cognitive impairment (MCI)^10^ and dementia (D)^11^ in recent cohort studies, such as the Alzheimerʼs Disease Neuroimaging Initiative^12^, ^13^, ^14^ and the Dominantly Inherited Alzheimer Network.^15^ The numbers of participants, mean age, sex, and total and subclassified domain scores of CogEvo, MMSE scores and CDT scores are described in Table 1. Each score of the CogEvo, MMSE and CDT was also assessed according to the cut‐off score groups of the MMSE.

**Figure 2:**
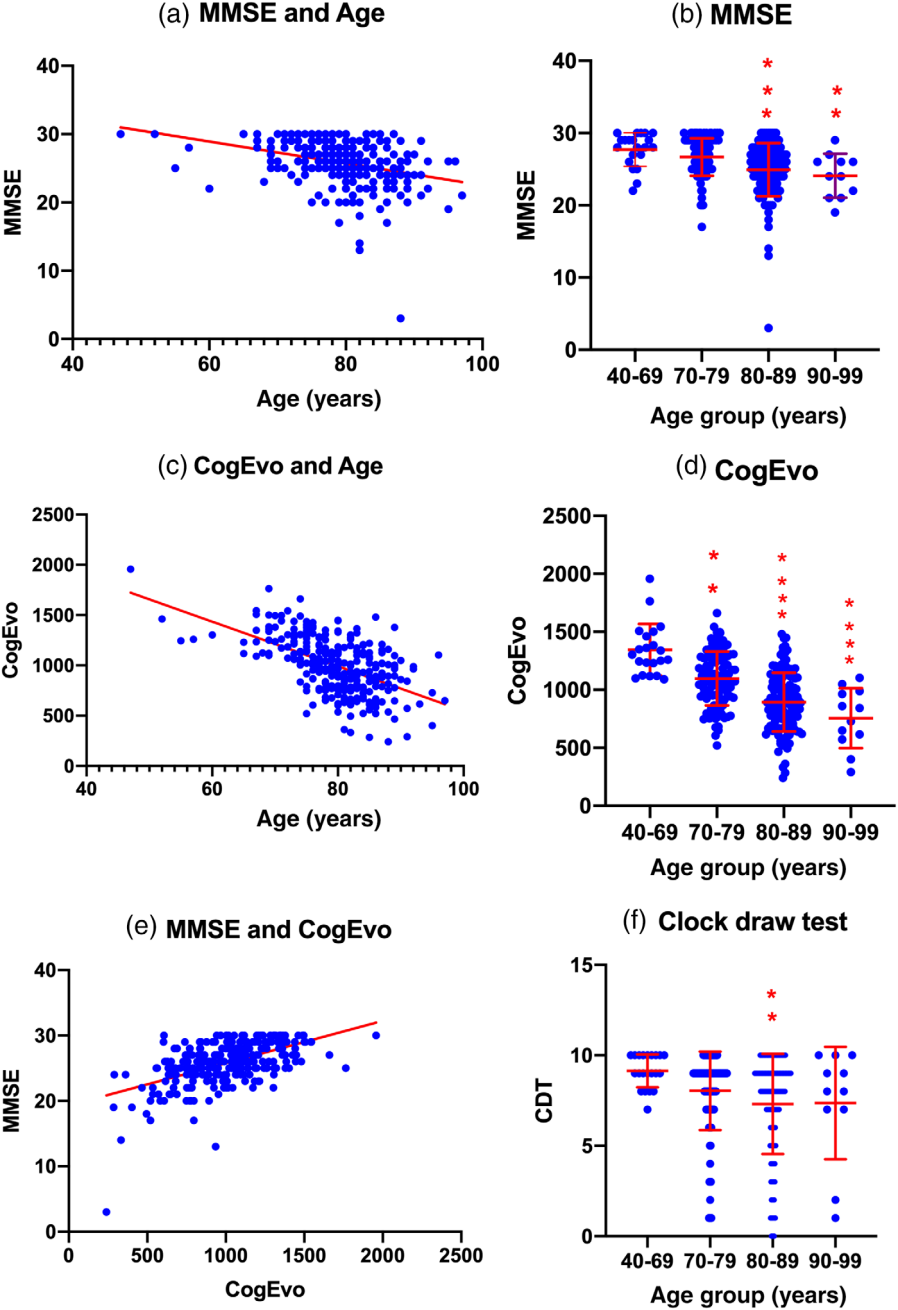
Association studies among the Mini‐Mental State Examination (MMSE), CogEvo, clock drawing test and age. **P < 0.005; ***P < 0.0005; ****P < 0.0001.

Then, we analyzed the association of total CogEvo scores and MMSE scores, with age. Both MMSE scores against age (y = −0.1602*X + 38.53, *r* ^2^ = 0.1115, *P* < 0.0001; Fig. 2a) and total CogEvo scores against age (Y = −22.19*X + 2766, *r* ^2^ = 0.3011, *P* < 0.0001; Fig. 2c) showed significant negative correlations. MMSE scores and total CogEvo scores showed a significant positive correlation (Y = 0.006471*X + 19.30, *r* ^2^ = 0.2973, *P* < 0.0001; Fig. 2e). Ceiling effects were noted in the association between MMSE and total CogEvo scores, and between MMSE scores and age because of the upper 30 points in the MMSE. However, these limitations were not observed in the linear regression curve between CogEvo scores against age (Fig. 2c).

We also compared the MMSE scores, total CogEvo scores and five subscore items of the CogEvo, such as orientation, attention, memory, executive function and spatial cognition, and CDT scores in the four age‐dependent groups. Total scores of the MMSE showed significant decreases in group 3 (*P* = 0.0003) and group 4 (*P* = 0.0028; Fig. 2b) compared with those of group 1. Total scores of the CogEvo test were significantly decreased in the corresponding age groups (group 2: *P* = 0.0029; group 3: *P* < 0.0001; and group 4: *P* < 0.0001; Fig. 2d). However, the CDT only showed significance in group 3 (*P* = 0.0025; Fig. 2f) compared with group 1.

**Figure 3:**
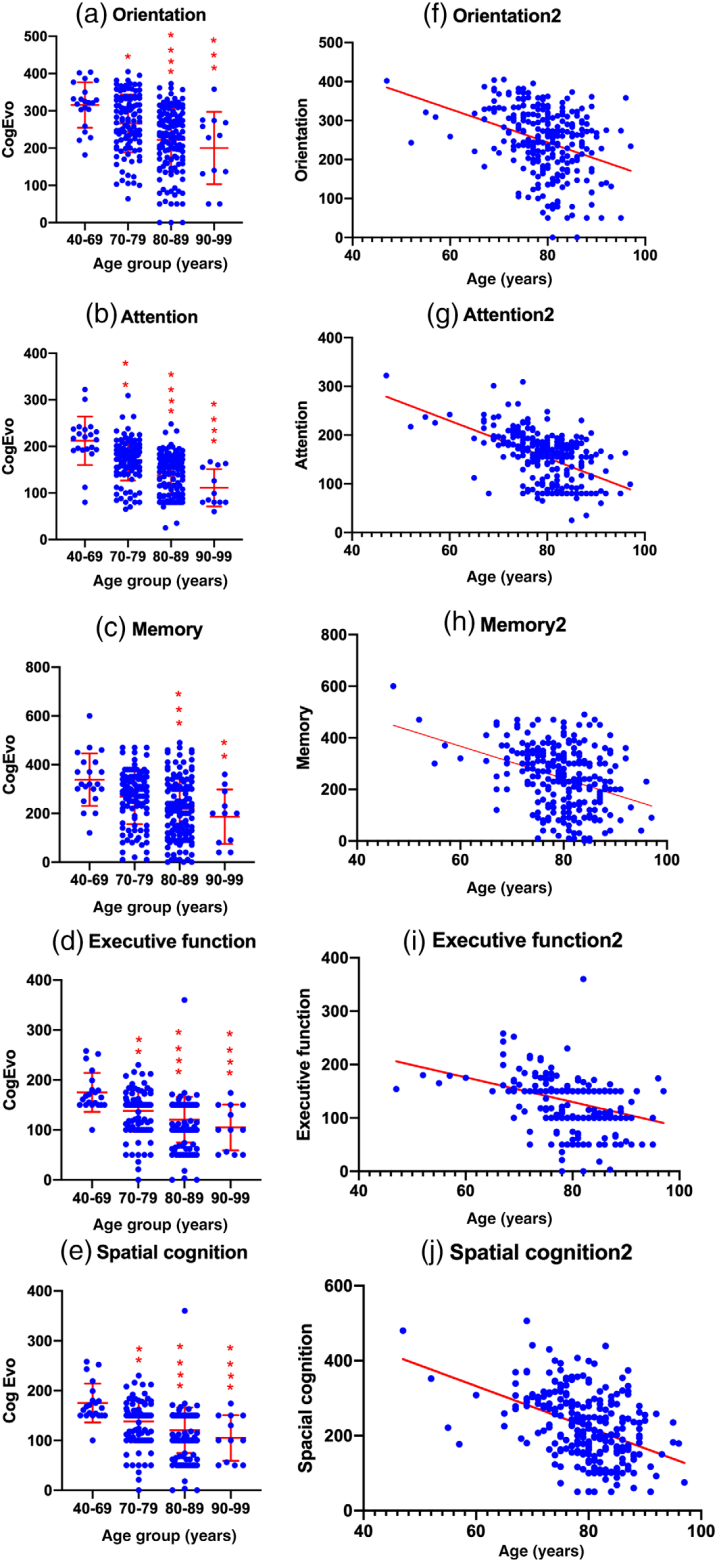
Age‐related decline of scores in CogEvo. *P < 0.05; **P < 0.005; ***P < 0.0005; ****P < 0.0001.

In the orientation item of the CogEvo test, a significant decline in scores was observed in group 2 (*P* = 0.0297), group 3 (*P* < 0.0001) and group 4 (*P* = 0.0009) compared with group 1 (Fig. 3a). In the attention items, a significant decline was shown in group 2 (*P* = 0.0033), group 3 (*P* < 0.0001) and group 4 (*P* < 0.0001; Fig. 3b). In the memory domain, significance was recognized in group 3 (*P* = 0.0002) and group 4 (*P* = 0.0031; Fig. 3c). Scores of executive functions also showed a significant decrease between group 2 (*P* = 0.0011), group 3 (*P* < 0.0001) and group 4 (*P* < 0.0001) (Fig. 3d). In spatial cognition items, a significant decline was observed in group 2 (*P* = 0.0011), group 3 (*P* < 0.0001) and group 4 (*P* < 0.0001; Fig. 3e). We also carried out a simple linear regression analysis between age and five subscore items of the CogEvo test. All subscore items also showed a significant linear correlation with age. Respective regression coefficients were −4.276 in orientation, −3.805 in attention, −6.259 in memory, −2.308 in executive function and −5.539 in spatial cognition. The coefficient of determinations (*r* ^2^) were 0.1194 in orientation, 0.2799 in attention, 0.1262 in memory, 0.1208 in executive function and 0.1812 in spatial cognition. These findings suggested that all five subscore items decreased according with age; however, attention subscores were more affected by the effect of age compared with the other four subscore items (Fig. 3f–j).

**Figure 4:**
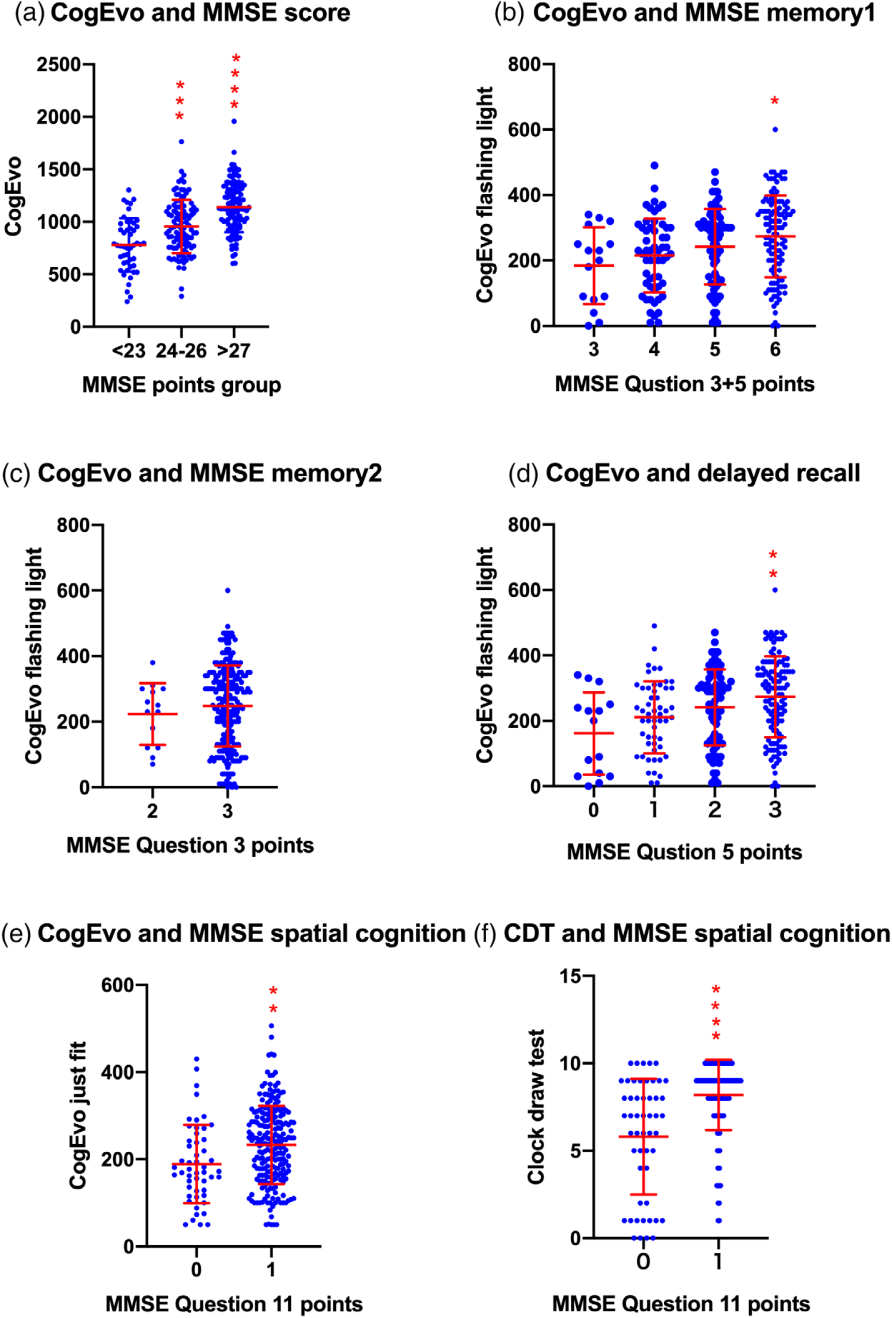
Association study of memory and spatial cognition in the CogEvo test and clock drawing test (CDT). *P < 0.05; **P < 0.005; ***P < 0.0005; ****P < 0.0001. MMSE, Mini‐Mental State Examination.

Then, we compared the subscale scores of the subclassification groups and domains of the CogEvo and MMSE tests. Total CogEvo scores were significantly increased in the 24–26 points group (*P* = 0.0002) and the >27 points group (*P* < 0.0001) compared with the <23 points group. Significance was observed in the 27 points group compared with the between 24 and 26 points group (*P* < 0.0001; Fig. 4a). To further study the memory domains, scores of the CogEvo flashing light and scores for questions 3 + 5 in the MMSE were analyzed, showing a significant increase in CogEvo scores between MMSE scores of 3 points and 6 points (*P* = 0.0441), and between 4 points and 6 points (*P* = 0.0131; Fig. 4b). A separate analysis showed no significant differences between 2 and 3 points for the MMSE question 3 (Fig 4c). However, significances were recognized between 0 and 3 points (*P* = 0.0091) and 1 and 3 points (*P* = 0.007) for MMSE question 5 (Fig. 4d), suggesting the CogEvo flashing light test reflected more delayed recall function memory items than those of the registration function. To analyze spatial cognition items, associations between MMSE question 11, CogEvo same shape and CDT were examined. Both item scores showed a significant relationship with MMSE question 11 for CogEvo same shape (0–1 point: *P* = 0.0002; Fig. 4e) and in the CDT (0–1 point: *P* < 0.0001; Fig. 4f).

Finally, we examined the sensitivity and specificity of the CogEvo total scores using receiver operating characteristic analysis for discriminate CU, MCI and D cut‐offs defined by MMSE scores. Setting 809 points of the total scores of the CogEvo test, the D and MCI groups were discriminated at a sensitivity of 70% and specificity of 60%. At 995 points of the CogEvo test, sensitivity of 78% and specificity of 54% were observed between the MCI and CU groups. A sensitivity of 85% and specificity of 70% at 1018 points were obtained for comparisons between the D and CU groups.

## Discussion

MMSE is the worldwide established screening neuropsychiatry test that consists of orientation of time and place, memory registration and recall, attention and calculation, language, and executive and visuospatial proficiency assessment.^1^ Testing took 5–10 min, and the cut‐off value of 23 out of 24 discriminated moderate‐to‐severe cognitive impairment.^2^, ^3^ However, scores were highly dependent on age and education level.^16^ Population‐based norms have been reported from the age of 6 years to >85 years,^17^, ^18^ and the natural decline of MMSE scores is 3–4 points per year in Alzheimerʼs disease.^16^, ^19^ MMSE is a basic variable for observational study, such as Alzheimerʼs Disease Neuroimaging Initiative^12^, ^13^, ^14^ and Dominantly Inherited Alzheimer Network,^15^ and is one of the end‐points of intervention study of disease‐modifying drugs. For this reason, a comparison between the MMSE and CogEvo is meaningful.

The total examination time for the MMSE and CogEvo test is almost the same; however, the machinery instructions for the task and automatic score calculation seem to be easier and more consistent compared with the MMSE. Both total scores of MMSE and CogEvo test showed age‐dependent cognitive decline (Fig. 2). Ceiling effects at 30 points in the MMSE decreased the usefulness for detecting slight age‐related cognitive impairment (Fig. 2a,e). As MMSE was developed to discriminate dementia from normal behavior,^1^, ^2^ it suggests difficulty in recognizing slight cognitive impairment. In contrast, the CogEvo test was shown to detect these slight age‐related cognitive impairments clearly (Fig. 2c,d), suggesting that CogEvo is feasible for evaluating age‐related or preclinical cognitive decline from 65 years onwards in cohort studies or preclinical intervention trials. Further analysis in the subclassified domain of cognition showed that CogEvo also showed significant age‐related cognitive decline in orientation, attention, memory, executive function and spatial cognition (Fig. 3). However, the CDT did not show a clear significance and ceiling effects. The CDT is considered to be a test for discrimination between normal behavior and dementia, similar to the MMSE. Thus, CogEvo seems to be a sensitive test battery for age‐related cognitive decline.

These findings suggest that the CogEvo test might also be feasible for preclinical observation studies or clinical trials for the prevention of dementia. In the MMSE, the range for the CU participants and participants with MCI was 24–30, and for mild Alzheimer's disease dementia the range was 20–26.^12^, ^13^ The CDR score and education duration‐dependent delayed recall score of the logical Memory II subscale for Wechsler Memory Scale‐Revised were additional criteria for discernment of CU, MCI and mild Alzheimer's disease dementia.^10^ For this reason, we validated whether the CogEvo test could separate cut‐off points of 24 and 26 in MMSE scores. As shown in Figure 4a, CogEvo significantly discriminated these groups with cut‐offs at 780.4 points, and a sensitivity of 78% and specificity of 54%, and at 1128.1 points with a sensitivity 70% and specificity of 60%, suggesting that these cut‐offs also exist in the CogEvo total points range 0–2500 points.

Then, we further validated memory and the spatial cognition domain of CogEvo in detail. In comparisons between registration and recall functions of memory domain, CogEvo results were correlated with delayed recall more than registration (Fig. 4b–d). Both CogEvo and CDT significantly responded to MMSE question 11; however, a wide range of scores for the CogEvo test without ceiling effects is considered more feasible for examining the spatial cognition domain.

As Alzheimerʼs disease and neurodegenerative dementia diseases develop cognitive impairment after a very long preclinical period, discriminating physical age‐dependent declines in cognitive functions is very difficult, and therefore there is meaning in inventing a neuropsychiatry battery of the tests for evaluating age‐related cognitive decline. Clinical trials on disease‐modifying therapy are also changing ideas on the prevention in preclinical states. For this purpose, many computer‐aided or iPad‐based test batteries, such as Cogstate^20^, ^21^ and CANTAB,^22^, ^23^, ^24^, ^25^ are emerging in this field. These batteries have been applied in cohort studies, such as the Dominantly Inherited Alzheimer Network. The present study used CogEvo, which is one of the recently introduced tests. A limitation of CogEvo is there have been few validation cohort studies and comparison studies with other kinds of PC‐based batteries. This is the first validation study. We therefore are planning validation of CogEvo in other international cohort studies and comparison studies with Montreal Cognitive Assessment, Cogstat, CANTAB and other tests.

## Disclosure statement

The authors declare no conflict of interest.
